# Fundamental Aspects of SPPS and Green Chemical Peptide Synthesis

**DOI:** 10.1002/psc.70013

**Published:** 2025-04-10

**Authors:** Stephen B. H. Kent

**Affiliations:** ^1^ Department of Chemistry University of Chicago Chicago Illinois USA

## Abstract

This perspective essay will briefly recount fundamental physicochemical properties of the peptide‐resin that have led to the almost universal use of stepwise solid phase peptide synthesis (SPPS) for the chemical synthesis of peptides. The essay discusses multiple aspects that must be addressed if we are to develop truly green chemical peptide synthesis. An optimal SPPS approach that retains the advantages inherent to polymer‐supported chemical synthesis, combined with convergent synthesis based on modern chemical ligation methods for the condensation of unprotected peptide segments, will be described as a path to green synthesis of peptides and their efficient manufacture. Only the most pertinent primary literature is cited.

## Graphical abstract

Proposed green chemical synthesis scheme for efficient preparation of a (hypothetical) 20 residue peptide. Aqueous SPPS employing N‐carboxyanhydrides (NCAs) minimal side chain protection, sample displacement mode HPLC purification and convergent condensation of unprotected peptide segments by native chemical ligation in aqueous solution.

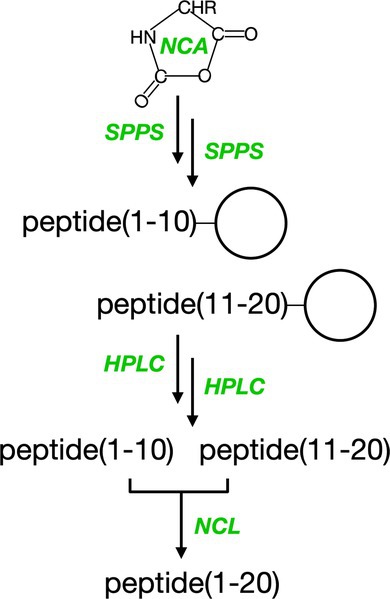

## 1 Introduction

The practice of chemical peptide synthesis is on the verge of radical changes, both in academic research and in the industrial manufacture of peptide therapeutics. There is a growing realization that synthetic protocols, solvents and reactants commonly used in solid phase peptide synthesis fall far short of the principles of green chemistry [1, 2, 3, 4, 5].

## 2 Solid Phase Peptide Synthesis

Solid phase peptide synthesis (SPPS), stepwise synthesis of peptide chains covalently attached to an insoluble resin support, was introduced by Bruce Merrifield in 1963 [6]. Merrifield's goal was to simplify and speed up the chemical synthesis of peptides [7]. After the initial skepticism and fierce opposition of the classical organic synthesis community, SPPS has become almost universally used for the chemical synthesis of peptides, both for academic research and in the commercial production of peptide therapeutics.

**SCHEME 1:**
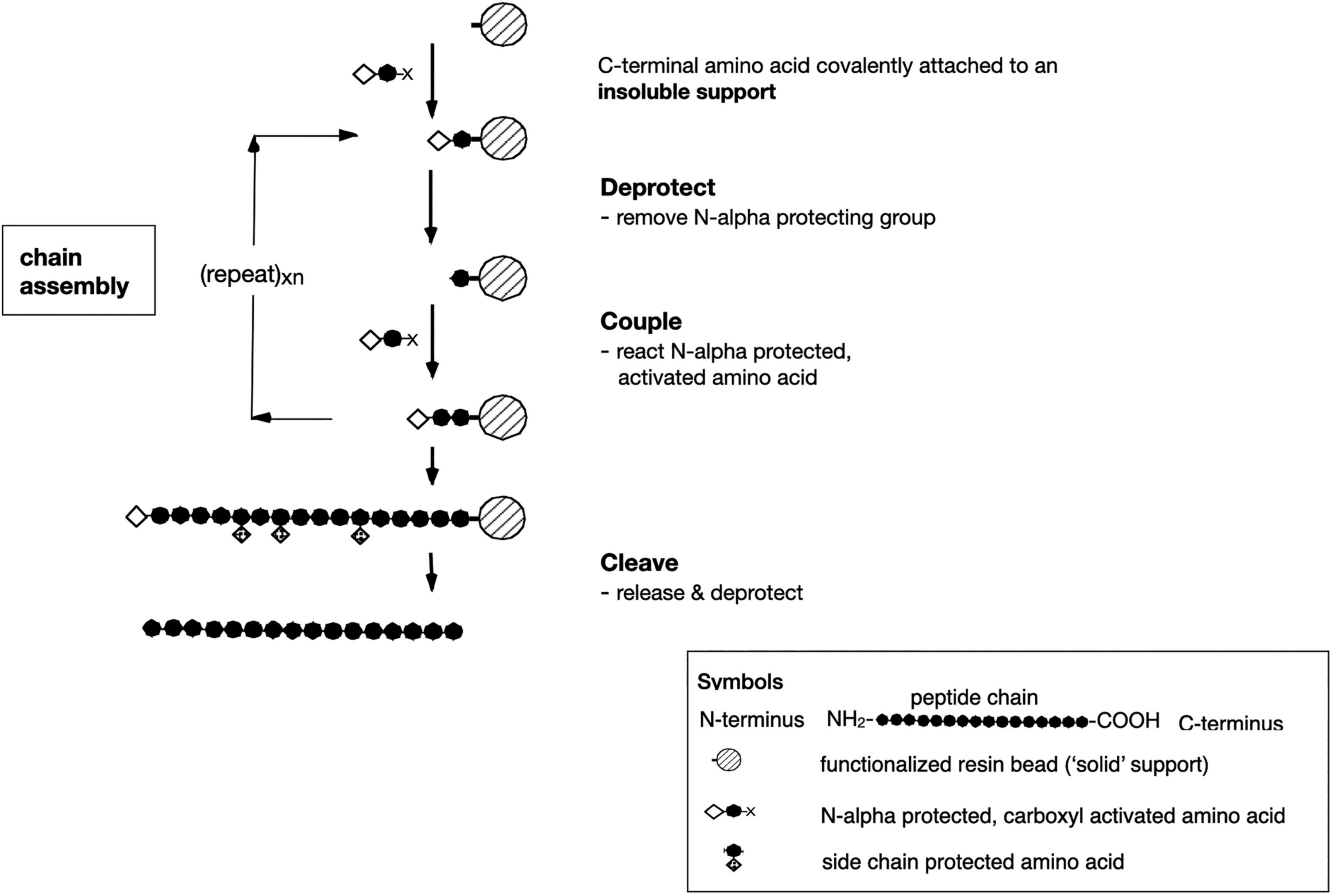
Diagrammatic representation of SPPS. This standard diagrammatic scheme for SPPS is misleading. Synthesis occurs *within* solvent‐swollen resin beads. There are ~10^14^ peptide chains covalently attached within a single ~50 μm diameter bead.

The principles of SPPS are well known and are illustrated in Scheme 1. The C‐terminal amino acid residue of the target peptide chain is covalently attached to crosslinked resin beads. Amino acids forming the target peptide chain are added one residue at a time in stepwise fashion by a repetitive set of chemical reactions. Standard solution organic chemistries are used for temporary protection of the α‐amino group, for α‐carboxyl activation, and for semi‐permanent protection of side chain functional groups during assembly of the peptide in SPPS.

**SCHEME 2:**
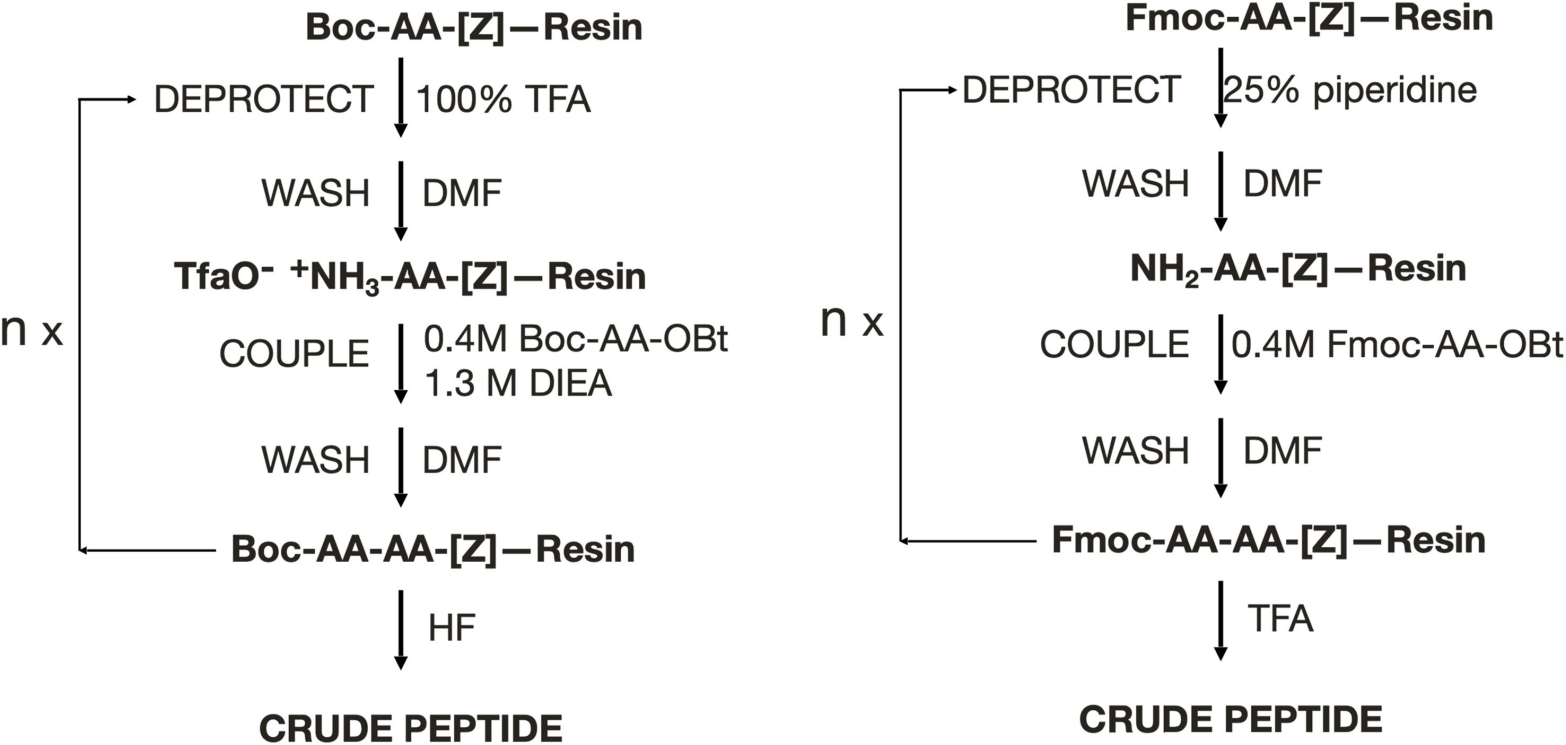
Typical SPPS protocols. (Left) Boc chemistry. (Right) Fmoc chemistry. The symbol [Z] represents the chemically labile linker moiety used to covalently attach the growing peptide chain to the polymeric resin beads.

Currently, Fmoc chemistry is most widely used for peptide synthesis by SPPS [8]. It employs the N^α^‐fluorenylmethyloxycarbonyl (Fmoc) protecting group that is removable by an elimination reaction under basic conditions, together with base‐stable, acid‐labile maximal protection of side chain functional groups. Boc chemistry SPPS, once the dominant SPPS chemistry but now more rarely used, is based on the graduated acid lability principle and uses the acid labile N^α^‐tertiarybutyloxycarbonyl (Boc) protecting group, together with side chain protecting groups that are resistant to the acidic conditions used to remove the Boc group but that can be removed by final treatment with a much stronger acid such as hydrogen fluoride [9]. A comparison of optimized versions of these two SPPS chemistries is shown in Scheme 2.

### 2.1 Is SPPS Green?

No. Neither Fmoc chemistry SPPS nor Boc chemistry SPPS comply with the principles of green chemistry (Box 1). As commonly practiced, SPPS uses toxic solvents and corrosive chemicals, generates high volume waste streams, and requires extensive purification of the crude peptides released from the resin in order to yield homogeneous molecular species of defined chemical structure.

Box 1 **Principles of green chemistry and features of SPPS that do not comply.**

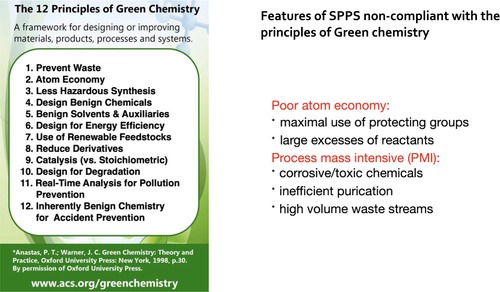

Fmoc chemistry SPPS is particularly egregious in terms of Atom Economy: the Fmoc group (mass 219 Da) itself is *twice* the average mass of amino acid residues in typical synthetic peptides; that means *only one third* of the mass of a typical Fmoc‐amino acid used in SPPS can end up in the peptide product. Furthermore, in SPPS typically a 3‐ to 5‐fold excess of each Fmoc‐amino acid is used in an effort to drive to completion the addition of each residue to the resin‐bound peptide chain.^1^

Commercial manufacture of peptides by SPPS is particularly poor in terms of ‘process mass intensity’, a key measure of overall synthetic efficiency that reflects the excesses of reactants used, the amounts of by‐products formed, and the volumes of solvents used in a synthesis [5]. Large amounts of solvent waste are generated during SPPS peptide chain assembly, and high volumes of solvents are used in preparative reverse phase HPLC purification of the crude peptide products obtained from stepwise synthesis.

## 3 SPPS Fundamentals

**FIGURE 1:**
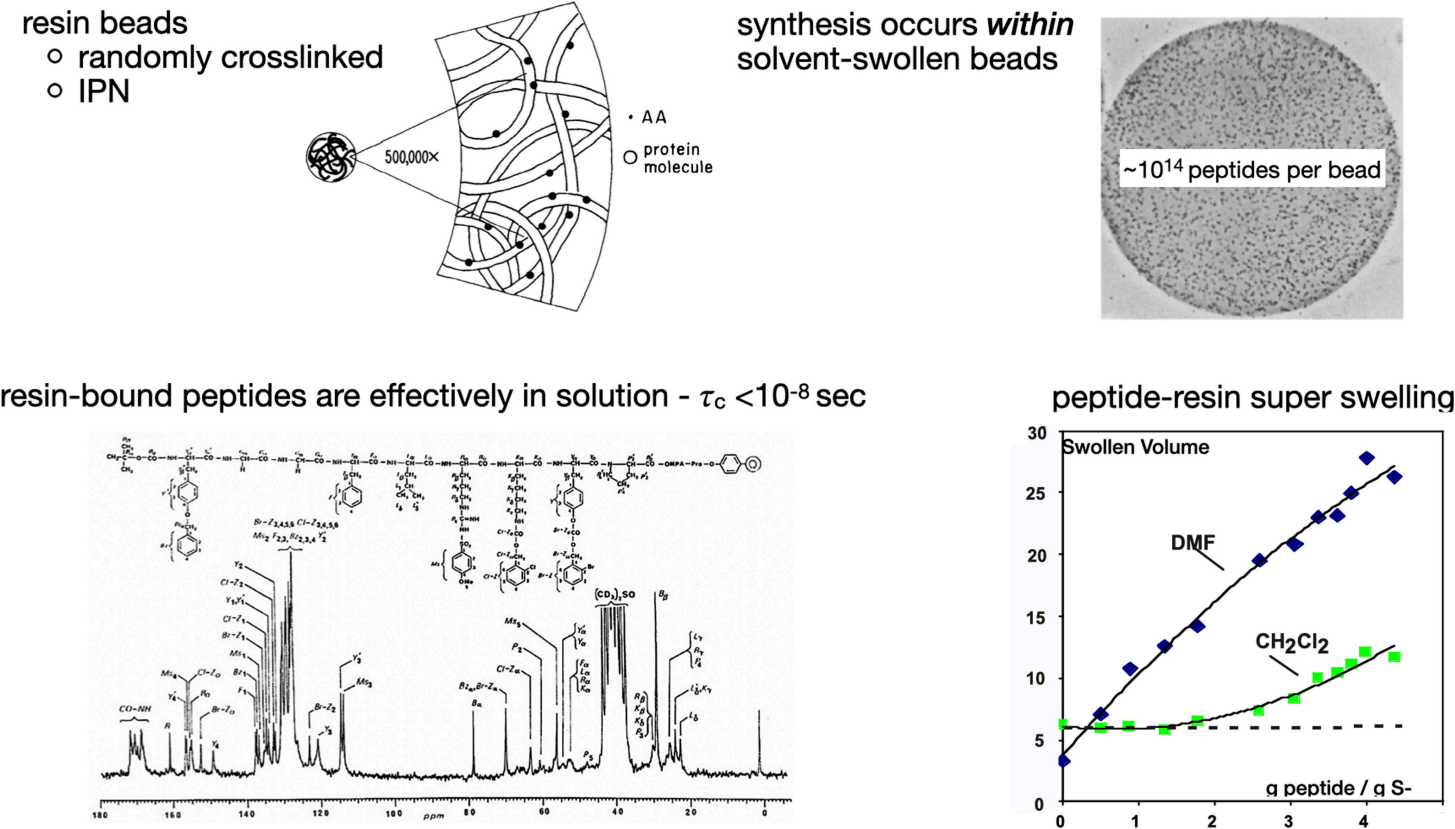
Essential features of SPPS. Top (left): resin beads are randomly crosslinked interpenetrating polymer networks (IPN). Top (right): synthesis occurs *within* the solvent‐swollen beads. Autoradiograph of a cross‐section of a labelled peptide‐resin bead. Bottom (left): narrow line ^13^C NMR spectra show that covalently attached peptide chains within the solvent‐swollen resin beads are effectively in solution. Bottom (right): free energy of solvation of lengthening synthetic peptide chains as the synthesis proceeds drives enhanced swelling of the resin beads.

If SPPS is so inefficient in terms of atom economy and process mass intensity, why is it so widely used for the chemical synthesis of peptides? The answer lies in fundamental physicochemical properties of the peptide‐resin during SPPS. Chemical synthesis of a peptide by SPPS starts with the C‐terminal amino acid residue of the target peptide molecule covalently attached to polymeric resin beads. Subsequent amino acids are added to that resin‐bound C‐terminal amino acid one at a time in stepwise fashion. The most commonly used resin is a suspension co‐polymer of styrene and 1% *meta*‐divinylbenzene (S‐DVB). In polymer chemistry terms, the resulting resin beads are a *randomly crosslinked interpenetrating polymer network (IPN)* (Figure 1, top left) [10]. Resin beads, fractionated to ~50 μm average diameter, imbibe organic solvents [11]. Solvation of the polymer chains within the resin bead leads to swelling until the decrease of the entropy of resin polymer chains because of covalent crosslinks balances the free energy of solvation of the polymer chains and prevents further volume increase [12]. Depending on the solvent used, S‐DVB resin beads swell to three to five times their dry volume in commonly used organic solvents.

Peptide synthesis occurs *within* the solvent‐swollen beads. At ~1 mmol peptide/g S‐DVB loading, there are ~10^14^ peptide chains within each ~50 μm diameter resin bead (Figure 1, top right) [9]. In the solvent swollen peptide‐resin beads, resin bound protected peptide chains are highly solvated; as measured by NMR methods, amino acid residues within the solvent swollen peptide‐resin beads display rotational correlation times Tau c ~ 10^−8^–10^−10^ s, similar to the correlation times of residues in protected peptide chains in free solution (Figure 1, bottom left) [13]. As SPPS chain extension proceeds, increased free energy of solvation of the growing linear peptide chains within the peptide‐resin beads drives increased swelling of the peptide‐resin (Figure 1, bottom right) [12].

In developing a truly green SPPS, it is essential to take into account the advantages that have led to its utility and to its near universal use for chemical synthesis of peptides. The advantages of SPPS, as commonly understood, are listed in Box 2. These are its simplicity, because of stepwise synthesis combined with purification by filtration; quantitative recovery of resin‐bound peptide product at every stage of a synthesis; and, use of general synthetic protocols that can also be applied to automated peptide synthesis.

Box 2 **Advantages of SPPS as commonly understood.**

● stepwise synthesis

● purification by filtration

● quantitative recoveries

● general protocols

● automation

### 3.1 Enhanced Solvation

In addition to being operationally simple and rapid, widespread adoption of SPPS derives from its near‐universal applicability to the chemical synthesis of moderately sized peptides of widely diverse amino acid sequences. This universality derives from fundamental physicochemical properties of the peptide‐resin that are not widely understood, nor is their importance appreciated. Most significantly, covalent attachment *within* solvent‐swollen polymeric resin beads leads to *enhanced solvation* of resin‐bound protected peptide chains, compared with the protected peptides in free solution in the same solvents [14].

**FIGURE 2:**
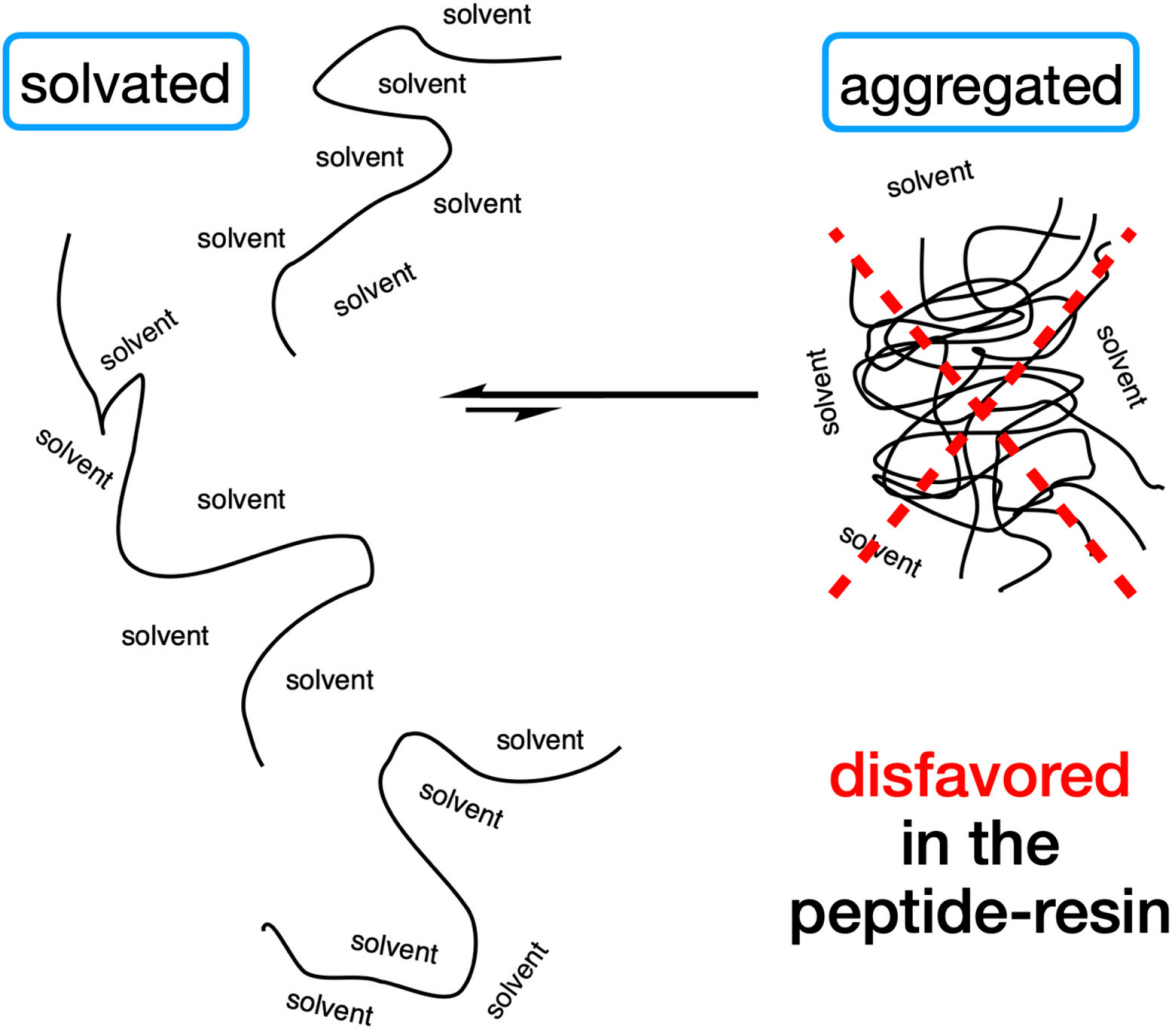
The extent of peptide solvation depends on the relative free energies of the two states shown above. Enhanced solvation of resin‐bound peptide chains compared to free solution arises because of the distinct chemical properties of the protected peptide chains and the resin polystyrene chains. Resin crosslinks prevent effective phase separation of the peptide and polystyrene chains with the resin beads [12].

Enhanced solvation of resin‐bound peptide chains originates from the *dissimilar* natures of the resin polymer and protected peptide chains, which *disfavours* the aggregated (non‐solvated) state. Furthermore, the resin polymer *crosslinks prevent phase separation* of the incipiently aggregating protected peptide chains from the polymer chains within a resin bead. The unfavourable thermodynamic consequences of these two effects strongly favour solvation of the resin‐bound peptide chains [12, 14] (Figure 2). Enhanced solvation of protected peptide chains within the solvent‐swollen resin beads is the *fundamental reason* for the efficacy and versatility of SPPS. The challenge is to devise an approach to green SPPS that retains this vital enhanced solvation phenomenon.

## 4 Green SPPS

Recent efforts to improve green aspects of SPPS have primarily focused on the use of more benign solvents in reduced amounts using Fmoc chemistry [15, 16, 17]. Although this has led to modest improvements, the impact of that approach is fundamentally limited. Other nongreen aspects of SPPS that have greater impact must also be addressed (Box 3). Green atom economy, minimizing the fraction of a protected amino acid reactant that ends up in the peptide product, requires minimal use of protecting groups together with minimal mass of protecting groups that are used, combined with minimal excess of protected amino acid in each peptide bond‐forming step. In addition to improved atom economy, more efficient methods for the purification of crude peptide products are necessary in order to reduce the excessive process mass intensity (total amount of waste generated, including the preparation of all reactants and product purification) inherent to SPPS as currently practiced. In the following several sections, optimization of each of these aspects of chemical peptide synthesis by SPPS will be addressed.

Box 3 **Optimal features of green stepwise SPPS.**

Atom economy:

● minimal use of protecting groups.

● minimal mass protecting groups.

● minimal excesses of reactants.

Process mass intensity:

● benign reactants & reagents.

● benign solvents.

● efficient purification.

### 4.1 Atom Economy

Conventionally, stepwise SPPS is carried out from the C terminal amino acid residue of the target peptide chain towards the N‐terminus, in order to minimize racemization [9]. Protection of the N^α^‐amino group of each carboxyl‐activated amino acid is required to prevent its oligomerization. For that reason, the mass of the N^α^‐amino protecting group has a major impact on atom economy. It is essential to minimize its mass.

**SCHEME 3:**
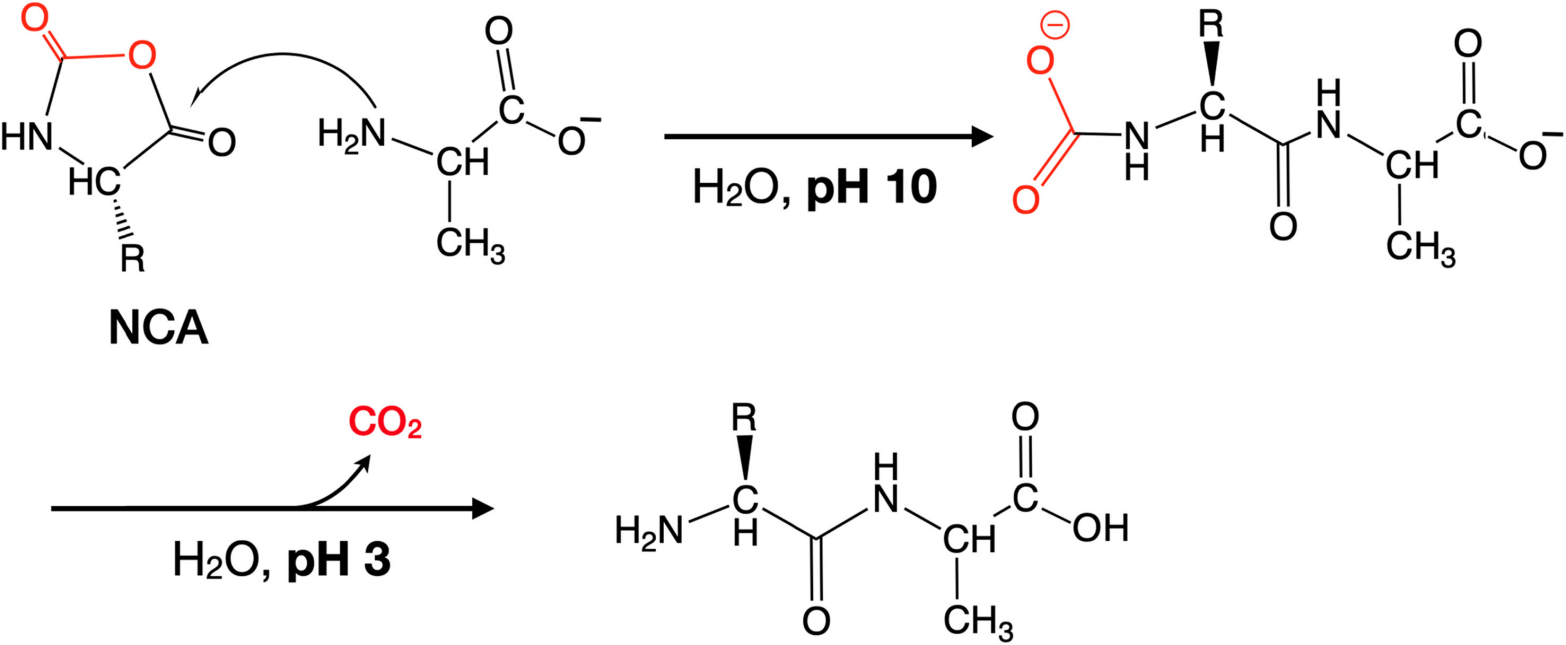
Using pH control to react an α‐amino acid N‐carboxyanhydride (NCA) to form a peptide bond in solvent water.

A promising candidate for a suitable N^α^‐protected form of amino acids in SPPS is the α‐amino acid N‐carboxyanhydride (NCA). NCAs have been known for more than 100 years and have been widely used for the preparation of amino acid homopolymers, and for a range of other applications in peptide‐based materials science [18, 19]. NCAs are N^α^‐protected and at the same time are carboxyl‐activated due to formation of a five‐membered ring in which the amino acid α‐carboxyl is attached to the α‐amino group of the same amino acid molecule by insertion of a carbonyl group (Scheme 3).

Peptide bond formation by reaction of an NCA with another amino acid is rapid and releases CO_2_ as co‐product, resulting in very high atom economy: loss of CO_2_ in the reaction amounts to just 44 Da mass. Thus, for peptide bond formation using the N^α^‐carboxyanhydride of an amino acid of average mass ~120 Da, the inherent atom economy will be greater than 70%. Furthermore, NCAs are self‐activated—no additional activating agent would be required for peptide bond formation during SPPS. Importantly, a promising efficient green preparation of α‐amino acid N‐carboxyanhydrides has recently been reported [20].

To date, application of NCAs to the controlled synthesis of peptides of defined amino acid sequence has been limited. Notably, starting in 1966 the Hirschmann group at Merck reported a series of systematic investigations of the use of NCAs for the synthesis of peptides in aqueous solution by means of pH control to prevent polymerization of the NCA reactant (Scheme 3) [21, 22, 23]. They reported rapid peptide bond formation in aqueous solution with near‐quantitative yields, using minimal excess amounts of the NCA reactants, with sparse side‐chain protection. Racemization was not observed. Several years later the same group reported use of NCAs in aqueous solution in the synthesis of minimally protected peptide segments comprising amino acid residues 21–124 of the enzyme ribonuclease S; 40% of the peptide bonds in those segments were formed through the use of NCAs [24].

**SCHEME 4:**
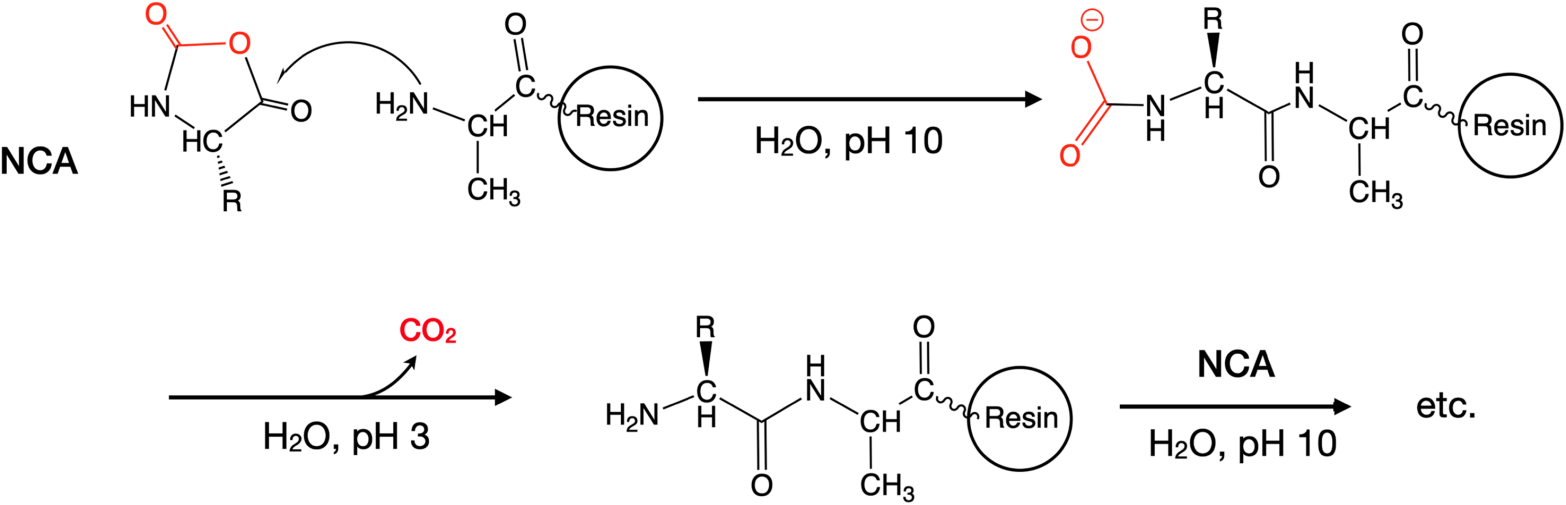
NCA‐based SPPS [24].

Peptide bond formation using NCAs can be carried out under mild aqueous reaction conditions that permit minimal use of side chain protecting groups, further enhancing the atom economy of peptide synthesis using these reactants. In Fmoc chemistry SPPS as normally carried out at the present day, side chain functional groups of 10‐to‐12 of the 20 common genetically encoded amino acids require a protecting group in order to prevent side reactions during peptide synthesis. In contrast, reported use of NCAs to synthesize peptides in aqueous solvents only required protection of the side chain functional groups of histidine, lysine and cysteine residues [21, 22, 23]. More recently, the Gentilucci group at the University of Bologna reported the use of NCAs for stepwise SPPS on ChemMatrix polyethyleneglycol resin, in aqueous buffer with pH control to prevent polymerization of the NCA reactant [25] (Scheme 4). This interesting study constitutes a preliminary ‘proof‐of‐concept’ for the use of NCAs for green solid phase peptide synthesis.

### 4.2 Aqueous‐Organic Solvents Compatible Resin

**SCHEME 5:**
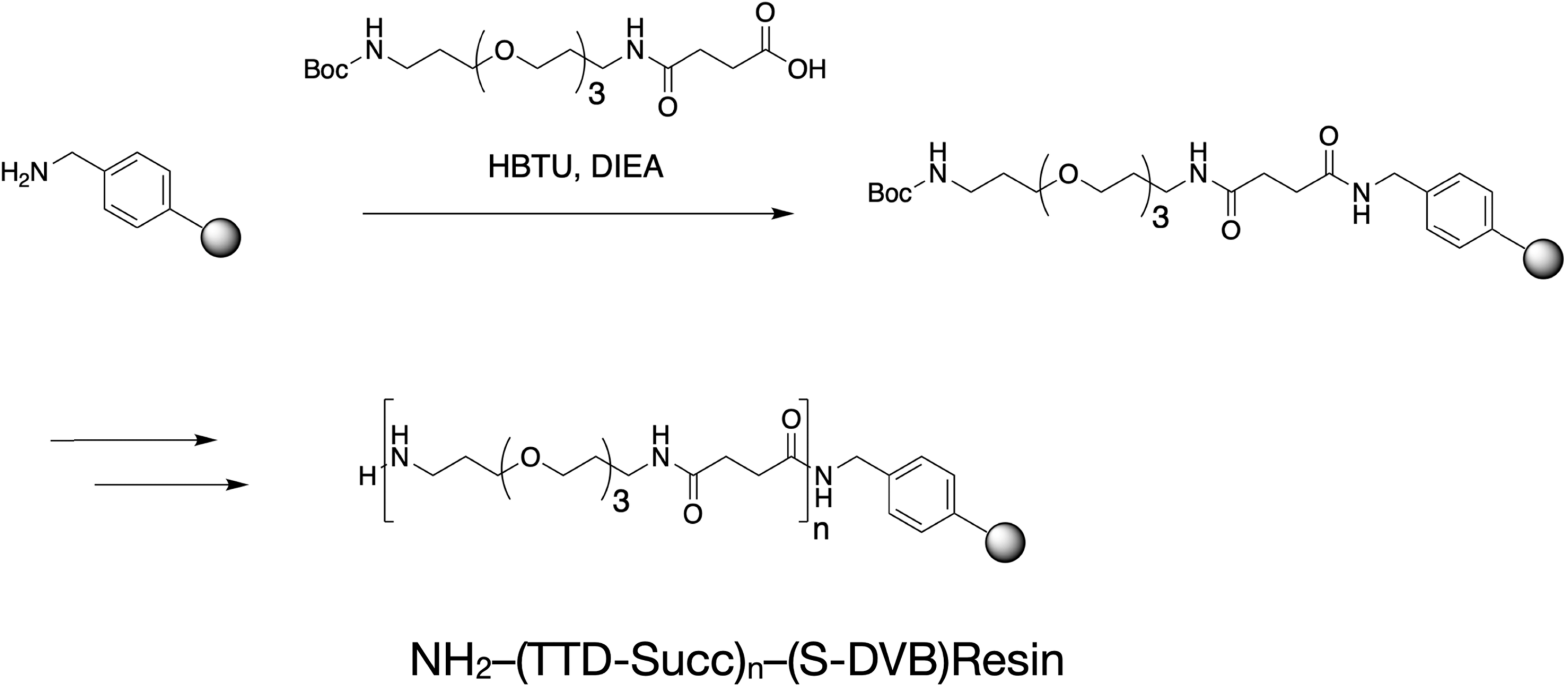
Preparation of NH2–(TTD‐Succ)_n_–(S‐DVB)resin from aminomethyl‐(S‐DVB) resin [26].

SPPS based on the use of amino acid N‐carboxyanhydrides requires a resin support compatible with both aqueous and organic solvents.^2^ The NH_2_–(TTD‐Succ)_n_–(S‐DVB) is just such a resin. Importantly, it can be prepared in chemically defined form and in large quantity from readily available starting materials: succinic anhydride; the epoxy curing agent 4,7,10‐trioxa‐1,13‐tridecanediamine (TTD); and aminomethyl‐copoly(S‐DVB) resin [26] (Scheme 5).

NH_2_–(TTD‐Succ)_n_–(S‐DVB) resin has properties similar to those of PEG‐poylstyrene resin [27]. It can be used with organic solvents for covalent attachment of the C‐terminal amino acid residue to the resin support and is compatible with the use of aqueous solvents for stepwise chain extension in SPPS. A high loading of the primary amine group to which the peptide chain is attached, typically ~1 mmol/g S‐DVB, is used in order to maximize the concentration of the resin‐bound peptide reactant during chain extension peptide bond‐forming reactions. Precursor aminomethyl‐(S‐DVB) resin is prepared by reaction of S‐DVB resin beads with N‐hydroxymethylphthalimide under strong Lewis acid conditions [28]. Importantly, these acidic reaction conditions scavenge the residual functional groups that inevitably exist in commercial S‐DVB resin beads, by reaction of those functional groups with the mildly activated para‐alkyl substituted phenyl rings that are omnipresent in S‐DVB resin beads [29, 30].

In order to avoid the possibility of side‐reactions reintroducing other extraneous functional groups, solution chemistry should be used to first prepare the C‐terminal amino acid of the target peptide in a form that incorporates the chemically cleavable ‘linker’ functionality for release of the product peptide from the resin support [31, 32]. The preformed N^𝛂^‐protected amino acid‐(linker)‐COOH unit is covalently attached to the NH_2_–(TTD‐Succ)_n_–(S‐DVB)resin by straightforward amide bond formation using minimal excess reactant at a high concentration, insuring minimal by‐product formation on the loaded resin beads.

### 4.3 Minimal Amounts of NCA Reactants at Maximal Concentration

During stepwise extension of the polypeptide chain, a *minimal excess amount* ^3^ of each NCA reactant should be used at *maximum* practical *concentration*. This will insure efficient peptide bond formation, rapid reaction, high yields and improved atom economy. Past experience with Boc chemistry SPPS using resin loadings of ~1 mmol/g S‐DVB suggests that at concentrations of activated amino acid approaching 0.5 M, as little as a 20% molar excess leads to near quantitative peptide bond formation in a few minutes.

### 4.4 Minimal Side Chain Protection

Under appropriate NCA peptide bond‐forming reaction conditions in aqueous solvent, it will be necessary to protect only the side chain functional groups of histidine, lysine, and cysteine residues [21, 33]. Chain extension will require just the NCA itself and benign other reactants: namely, low concentrations of hydroxide ions during peptide bond formation, and similarly low concentration of protons during release of carbon dioxide from the N‐terminus of the resin bound peptide after addition of each amino acid residue.

### 4.5 Final Deprotection and Cleavage From the Resin

Once the target peptide chain has been assembled on the resin, any side chain protecting groups are removed and the product peptide is released from the resin by suitable chemical treatment(s). Both these steps should be performed under green reaction conditions, such as photolysis [34], nucleophilic cleavage or simple elimination reactions. Note that use of trifluoroacetic acid does not comply with the principles of green chemistry [35].

### 4.6 Low Process Mass Intensity Purification of Synthetic Peptides

Stepwise SPPS is carried out without purification of the resin‐bound peptide during synthesis. Consequently, the crude peptide product released from the resin will contain all resin‐bound peptide by‐products that have been formed during assembly of the target peptide chain. In SPPS of peptides containing ~15 or more amino acids, large numbers of by‐products will be formed each present at low level in the crude product. These by‐products include *deletions*, where one or more internal amino acids are missing from the peptide chain; *terminations*, resulting in shorter peptide chains with free or blocked N‐terminals; and, *chemical modifications* of the resin‐bound peptide that have occurred during the synthesis [9]. Often, the total amount of by‐products will exceed the amount of target peptide in the crude product. Most of these by‐products will have structures and hence properties that are closely similar to those of the target synthetic peptide.

Such complex product mixtures represent a significant purification challenge [36, 37]. A useful first step is tag‐assisted isolation of the peptide chain after cleavage from the resin [38]. Covalent tag‐assisted purification can be used to remove any N‐terminally blocked by‐products, and to recover the partially purified target peptide product with minimal solvent usage and will substantially reduce the need for time‐consuming lyophilization steps with their high energy demands.

### 4.7 Samples Displacement Mode Preparative HPLC

Final purification, to remove the remaining by‐products formed during chain extension, which include isobaric isomerizations such as N‐to‐O acyl shifts and racemization, by‐products that have masses distinct from the target peptide from formation of succinimide rings or hydrolysis of amide side chains, and a variety of other covalent modifications, should be carried out using ‘sample displacement mode’ preparative reverse phase HPLC^4^ [39, 40, 41, 42]. High amounts of crude peptide are loaded onto a reverse phase support under isocratic conditions to form a ‘displacement train’, a set of non‐overlapping regions each of which contains an individual peptide component that pushes the next modified peptide or the target peptide ahead of it off the HPLC column. Displacement mode purification not only dramatically reduces solvent consumption but also results in higher recoveries and greater purity of SPPS peptide products.

### 4.8 Characterization of Synthetic Peptides

The goal of chemical peptide synthesis is to provide a *single molecular species of defined covalent structure* [43, 44]. When reverse phase HPLC is used to purify the target peptide, it is not meaningful to use analytical HPLC or analytical LCMS to verify homogeneity of the purified peptide. Homogeneity of the purified synthetic peptide should be established using at least two high resolution analytical methods that operate on distinct separation principles, and that both differ from the separation method used to purify the peptide [45]. Examples of suitable analytical methods include *direct infusion* of the purified peptide in analytical electrospray mass spectrometry (MS) [46], which will reveal any peptide components of distinct mass that are present, and capillary electrophoresis or isoelectric focusing (IEF) both of which separate peptide coproducts based on their charge properties [47].

Once satisfactory homogeneity of a purified synthetic peptide has been established, its covalent structure should be verified. Confirmation of the expected mass of the peptide measured by electrospray MS is a *necessary*, but is NOT a *sufficient* verification of covalent structure [45]. The amino acid sequence of the peptide should be experimentally confirmed. A simple and effective way of experimentally determining the amino acid sequence of a synthetic peptide prepared by stepwise SPPS is MALDI‐TOF time‐of‐flight MS ‘ladder sequencing’ of pooled samples taken after addition of each amino acid during chain assembly [48]. Alternatively, the amino acid sequence can be verified using a ladder of sequence‐determining fragments generated by post‐synthesis chemical treatment of the synthetic peptide [49].

## 5 Green Chemical Manufacture of Therapeutic Peptides

Commercial manufacture of therapeutic peptides has requirements distinct from the chemical synthesis of peptides for research purposes. For scale manufacture of peptide therapeutics in kilogram and multikilogram amounts, product purity, process reliability and cost efficiency are paramount.

### 5.1 Convergent Chemical Synthesis

Convergent chemical synthesis is inherently more efficient than stepwise synthesis [50, 51]. In recent decades the potential greater efficiency of convergent synthesis of peptides has been lost sight of because of the simplicity and convenience of stepwise SPPS. Indeed, SPPS is often used in scale manufacture of peptide therapeutics because process development is straightforward. It is well established that convergent synthesis makes the most efficient use of starting materials and involves minimum exposure of each part of the target molecule to the synthetic reaction conditions, thus generating fewer by‐products [50]. Furthermore, isolation and purification of intermediate synthetic products, frequently decried as arduous and wasteful [52, 53, 54, 55], in reality can give both enhanced yields and greater purity of the final product when well performed [51].

### 5.2 Convergent Condensation of Unprotected Peptides

How can the advantages of convergent synthesis be realized in a more efficient Green manufacture of peptides by chemical means? The well‐known native chemical ligation (NCL) reaction enables covalent condensation of *unprotected* peptides to give a product that has a peptide bond at the ligation site [56]. Native chemical ligation itself is inherently green (Box 4) [2, 57].

Box 4 **Native chemical ligation—compliance with Green chemistry principles.**

(Compare with Box 1)

○ **Protecting groups** ‐ minimal/none.

○ **Solvent** ‐ water (w or w/o solubility modifiers).

○**Reaction conditions** ‐ benign: neutral pH.

○**Energy** ‐ reactions at/near room temperature.

○**Efficiency** ‐ minimal excesses of reactants.

○**Atom economy** ‐ complete (addition rxns)/very good (NCL).

○ **Products** ‐ degradable.

In academic research, aqueous 6 M guanidine hydrochloride is typically used as solvent in order to insure solubility of the reacting peptides. In process development for peptide manufacture using NCL, the amount of chaotrope can be minimized based on the solubility properties of the reacting peptide segments.

### 5.3 Convergent vs. Stepwise Synthesis

**SCHEME 6:**
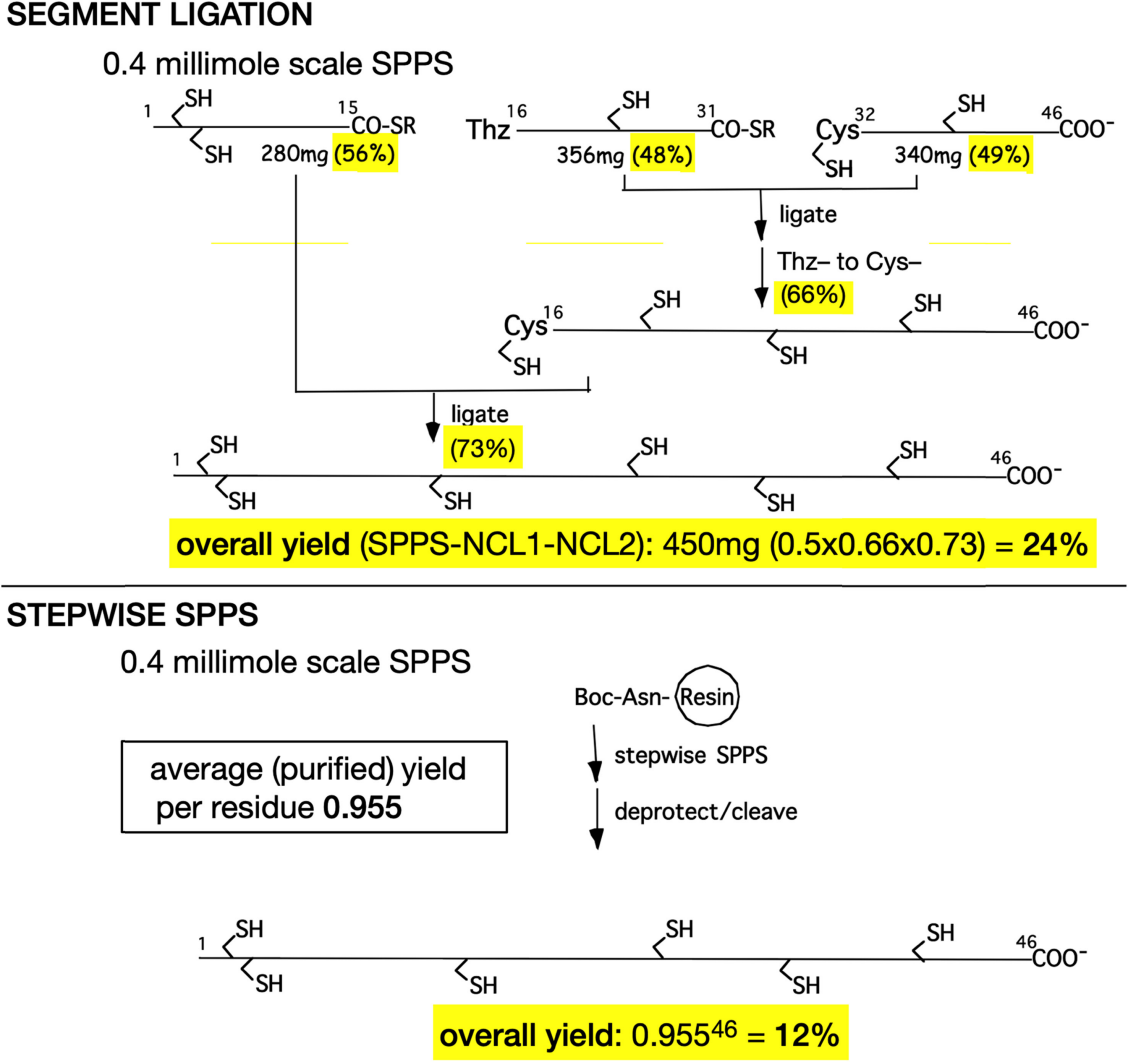
Convergent segment ligation vs. stepwise synthesis of the 46 residue peptide chain of Crambin. Purified yields are highlighted in yellow. For the short peptide segments, the isolated yields corresponded to an efficiency of 95.5% per amino acid residue. Note that 95.5% is NOT the stepwise SPPS coupling yield (typically > 99.5%)—rather, it is the *overall efficiency per amino acid residue*, calculated from the final SPPS yield including purification of the peptide to homogeneity. Yields of the NCL condensation steps are the average of three repetitions of the synthesis [58].

The advantages of convergent condensation of unprotected peptides by native chemical ligation are illustrated by the yield obtained in synthesis of the 46 amino acid residue polypeptide chain of the plant protein Crambin by convergent condensation of three synthetic peptide segments with purification of intermediate synthetic products, compared with the yield obtained by stepwise SPPS at the same average efficiency per amino acid residue [58] (Scheme 6).

As illustrated by this example, well‐executed convergent synthesis by native chemical ligation of unprotected peptides, with isolation and purification of intermediates, can give both higher purity products and higher yields.^5^

### 5.4 Synthesis of Peptide Thioesters

The most versatile way to make peptide thioester building blocks is via peptide‐hydrazides [59]. Peptide hydrazides can be efficiently prepared [60], and converted to the peptide thioesters in aqueous solution under mild conditions via the Knorr pyrazole [61]. Furthermore, peptide hydrazides provide for fully convergent chemical condensations by NCL, especially from four unprotected peptide segment building blocks [62].

For the application of NCL, both in research and therapeutic manufacture wider commercial availability of (𝛽‐SH)Yaa amino acids is essential in order to enable condensation by NCL to be performed at a broad range of Xaa‐Yaa sites [63]. Efficient and flexible syntheses of a variety of 𝛽‐SH amino acids have been reported [64]. If required, rapid and quantitative desulfurization of ligated peptides has also been reported [57, 65].

Use of NCA‐based stepwise SPPS and efficient purification methods for the preparation of high purity peptide segments, followed by their condensation using native chemical ligation in aqueous solvents will provide a truly Green, more cost‐effective scale manufacture of peptide and small protein therapeutics. Even for research purposes, chemical synthesis of peptides greater than ~35–40 amino acid residues should be carried out by convergent condensation using native chemical ligation in order to obtain satisfactory yields of high purity peptide products [66, 67]. In addition to providing improved yields and higher purity, convergent synthesis is the most versatile route for preparation of analogues because each region of a target polypeptide chain is the same minimal number of synthetic steps to the product [50].

## 6 Summary and Conclusions

Box 5 **Green solid phase peptide synthesis.**

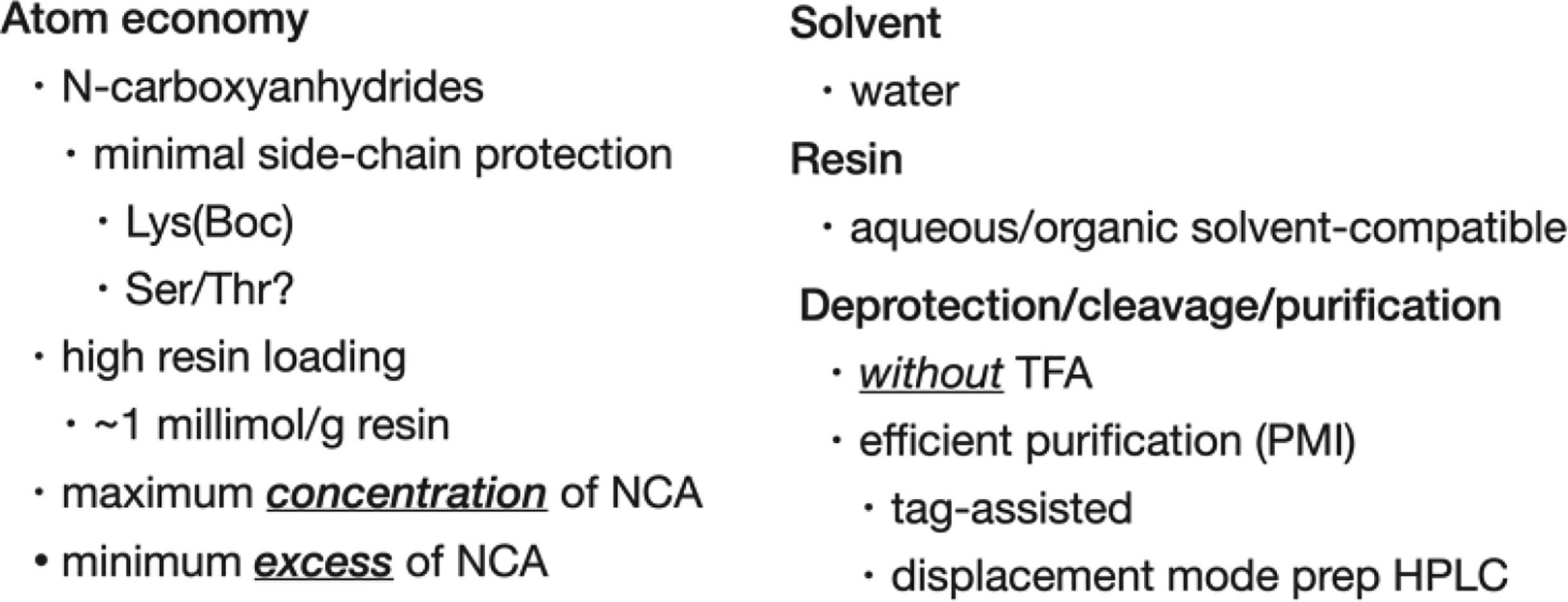

Green chemical synthesis of peptides as outlined in this essay is summarized in Box 5. More efficient manufacture of peptides and small protein molecules can be achieved by *convergent* native chemical ligation of unprotected peptide segments prepared by stepwise SPPS using amino acid N‐carboxyanhydrides in aqueous buffer and an aqueous‐compatible resin, combined with low process mass intensity and more effective peptide purification by sample displacement mode chromatography.

On a final note, the development of an optimized, robust green SPPS based on the use of N^𝛂^‐unprotected amino acid N‐carboxyanhydrides in aqueous solution will require both creativity and sustained, serious research in order to understand NCA properties and to optimize reaction conditions.^6^ Green preparation of NCAs, their sensitivity to moisture and appropriate storage conditions present challenges that will have to be addressed.

## Conflicts of Interest

The authors declare no conflicts of interest.

## Data Availability

The data that support the findings of this study are available on request from the corresponding author. The data are not publicly available due to privacy or ethical restrictions.

## Endnotes

^1^
Thinking in terms of *excess amount* of amino acid is incorrect. The *rate* of a bimolecular reaction, and hence the extent of reaction in a fixed time period, depends on the product of the *concentrations* of the two reactants; in this case, the activated amino acid and the resin‐bound peptide chain. It is essential to use the highest possible *concentration* of the activated amino acid, together with the highest practical ‘loading’ (mol peptide/g S‐DVB) of the protected peptide chain on the resin used for SPPS. Lower excess amounts of activated amino acids can then be used.

^2^
The PEG‐based resin ChemMatrix used by Gentilucci et al. [25] has ideal properties and is compatible with both organic and aqueous solvents. Unfortunately, it is no longer commercially manufactured.

^3^
Minimal excess relative to the molar amount of peptide‐resin.

^4^
Sample displacement mode should be contrasted with the more commonly used preparative HPLC method in which a gradient of increasing organic solvent concentration is used to separate peptide byproducts and elute the target peptide from the reverse phase support. Gradient elution uses large amounts of organic solvents, and significant overlap occurs at the edges of the Gaussian distributions of the components of the peptide mixture as they are displaced by the organic solvent gradient.

^5^
Mathematical treatments of stepwise versus convergent chemical synthesis have been published [50, 51]. An intuitive way to think about it is this: in stepwise SPPS, each new peptide bond‐forming reaction is performed on all the amino acid residues in the resin‐bound peptide and all the peptide byproducts formed to that point in the synthesis; in a convergent synthesis, each amino acid residue is exposed to fewer such reactions, and new peptide bonds are not being built on top of all existing byproducts, only the fewer byproducts in each smaller segment of the target sequence. This substantially reduces the total amount of byproducts in course of a synthesis. Furthermore, the smaller peptide segments and intermediate condensation products are more readily purified.

^6^
In that endeavour, direct real‐time monitoring of resin‐bound peptide products by MALDI‐TOF mass spectrometry will be useful [68].
